# An EST-based approach for identifying genes expressed in the intestine and gills of pre-smolt Atlantic salmon (*Salmo salar*)

**DOI:** 10.1186/1471-2164-6-171

**Published:** 2005-12-01

**Authors:** Heidi Hagen-Larsen, Jon K Laerdahl, Frank Panitz, Alexei Adzhubei, Bjørn Høyheim

**Affiliations:** 1 Norwegian School of Veterinary Science, Department of Basic Sciences and Aquatic Medicine. PO Box 8146 Dep., NO-0033 Oslo, Norway; 2 Biotechnology Centre of Oslo (BIO), University of Oslo, PO Box 1125 Blindern, 0317 Oslo, Norway; 3 Danish Institute of Agricultural Sciences, Department of Animal Breeding and Genetics, PO Box 50, DK-8830 Tjele, Denmark; 4 Rikshospitalet University Hospital, Department of Dermatology, NO-0027 Oslo, Norway

## Abstract

**Background:**

The Atlantic salmon is an important aquaculture species and a very interesting species biologically, since it spawns in fresh water and develops through several stages before becoming a smolt, the stage at which it migrates to the sea to feed. The dramatic change of habitat requires physiological, morphological and behavioural changes to prepare the salmon for its new environment. These changes are called the parr-smolt transformation or smoltification, and pre-adapt the salmon for survival and growth in the marine environment. The development of hypo-osmotic regulatory ability plays an important part in facilitating the transition from rivers to the sea. The physiological mechanisms behind the developmental changes are largely unknown. An understanding of the transformation process will be vital to the future of the aquaculture industry. A knowledge of which genes are expressed prior to the smoltification process is an important basis for further studies.

**Results:**

In all, 2974 unique sequences, consisting of 779 contigs and 2195 singlets, were generated for Atlantic salmon from two cDNA libraries constructed from the gills and the intestine, accession numbers [Genbank: CK877169-CK879929, CK884015-CK886537 and CN181112-CN181464]. Nearly 50% of the sequences were assigned putative functions because they showed similarity to known genes, mostly from other species, in one or more of the databases used. The Swiss-Prot database returned significant hits for 1005 sequences. These could be assigned predicted gene products, and 967 were annotated using Gene Ontology (GO) terms for molecular function, biological process and/or cellular component, employing an annotation transfer procedure.

**Conclusion:**

This paper describes the construction of two cDNA libraries from pre-smolt Atlantic salmon (*Salmo salar*) and the subsequent EST sequencing, clustering and assigning of putative function to 1005 genes expressed in the gills and/or intestine.

## Background

The number of known genes in Atlantic salmon (*Salmo salar*) is limited compared with the very large numbers known in mammals. However, the salmon is so important as an aquaculture species that a lot of effort has been put into acquiring knowledge of its genome compared to many of the other aquatic species. Although only 14 full-length genes are listed for Atlantic salmon at NCBI [1], there are 111 457-nucleotide sequences (September 2005), many containing complete cDNA sequences. The genome and biology of the Atlantic salmon are complex and the genome has not been well characterised. The salmonidae species underwent a genome duplication event about 25–100 million years ago [2,3]. This event resulted in a tetraploid genome, but since then the salmon has been gradually returning to the diploid condition. This process is still continuing, and the salmon now has a partially tetraploid genome. Although many genes have been lost after the duplication event [4], the fact that regions of the genome are duplicated and almost identical does complicate the hunt for genes in this species. It means that there may be four almost identical copies of a gene in the genome, but it is an open question whether all four copies are active. Furthermore, the Atlantic salmon goes through a process known as smoltification as a step in its maturation. The salmon spends the first part of its life in fresh water before migrating to the ocean, where it lives until returning to rivers to spawn. Smoltification [5] involves synchronised morphological, physiological and behavioural changes that enable the young salmon (parr) to survive in the ocean and to grow and migrate normally. Since smoltification occurs while the fish are still in fresh water, they are pre-adapted to the marine environment. Given this enormous change in both fish biology and environment, it seems reasonable to suggest that the pre-smolt expresses a different set of genes from the smolt. It is therefore important to identify the genes that are expressed both before and after the smoltification process and at each of the different developmental stages in order to obtain a complete set of genes expressed in the salmon genome. One strategy for identifying the genes expressed in specific life stages and tissues is to use expressed sequence tags or ESTs. These are short stretches of single pass sequences obtained from sequencing cDNA [6,7], and are widely used for gene discovery, mapping, polymorphism analysis, expression studies and gene prediction. Gene discovery methods using ESTs include hunting for new members of gene families in the same species (paralogues), for functionally equivalent genes in other species (orthologues), or even for alternatively spliced forms of known genes. ESTs are also used to predict or refine computational predictions of the location of genes in genomic DNA. Recently, a lot of effort has been put into acquiring ESTs from Atlantic salmon, and over 108,242 EST sequences (September 2005), mainly from smoltified fish, have been deposited in GenBank [8-11]The aim of the present study was to increase the number of ESTs available from pre-smolts in order to identify genes that are expressed in the early life stages of Atlantic salmon. We have focused on the gills and the intestine.

## Results

One of the goals was to accumulate cDNA libraries with large insert sizes. The average insert size was determined from 192 clones from each library fraction after digesting the clones with EcoRI/XhoI. The approximate size distribution for the libraries is given in table 1.

**Table 1:** Fraction sizes and number of sequences for the gills and intestine cDNA libraries

**Library fraction**	**Mean insert size**^**b**^	**Clones seq.**^**c**^	**Good seq.**^**c**^	**Unique seq.**^**c**^	**Annotated seq.**^**c**^
Gills 5	1,500 bp				
Gills 6	900 bp				
Gills 7	700 bp	4128	3015		
Intestine 4	1,300 bp				
Intestine 5	1,050 bp				
Intestine 6	730 bp	4128	3247		
Total				2974	1005

^a ^The numbers after the tissue type indicate when the cDNA was collected from the column, the lowest numbers being eluted first. The mean insert sizes are given in the second column

^b ^Shown in base pairs

^c ^Seq. is an abbreviation of sequence

A total of 4128 clones from each library were sequenced from the 5'direction. After basecalling and trimming of vector, contaminants and poor quality sequences, the number of sequences was reduced to 3015 for the gills and 3247 for the intestine. The trimmed sequences were clustered and assembled. After the combined clustering of the gill and intestine sequences, a total of 2974 unique sequences were left, of which 2195 were singlets and 779 were consensus sequences from contigs. Of these, 1491 showed similarity to known sequences after they were run through a combined blastx and blastn pipeline. A summary of the BLAST results is given in Table 2. All the sequence alignment results are accessible at the Salmon Genome Project website [11] in the libraries section (Data and Results > cDNA libraries > Gills, Intestine). All trimmed EST sequences have been submitted to GenBank. The accession numbers are [Genbank: CK877169-CK879929, CK884015-CK886537 and CN181112-CN181464]. The annotation of the clustered ESTs using GO terms is accessible through links at the Salmon Genome Project website [11] in the annotation section (Data and Results > Annotations > SGP Intestine-Gills). A shorter version is given in Table 3 for molecular function.

**Table 2:** Number of hits observed in the different databases

**Database**	**Hits**^**c**^	**%**^**d**^
PDB^a^	489	16
SwissProt^b^	1005	34
nr^b^	1276	43
nt^b^	961	32
no hits	1483	50

^a ^Threshold for a significant hit for blastx against PDB was defined as expectation value <10^-10^

^b ^Threshold for a significant hit for blastx against SwissProt and nr, and blastn against nr(= nt) was defined as expectation value <10–15

^c ^The number of significant hits observed in each databases

^d ^The number of significant hits observed given as a percentage

**Table 3:** Gene Ontology^a^(Molecular function) of the sequences with a significant blastx hit in SwissProt

**Molecular Function**^**b**^	**Nr of Hits**
%antioxidant activity	10
%glutathione-disulfide reductase activity	2
%peroxidase activity	5
%binding	505
%antigen binding	1
%carbohydrate binding	5
%cofactor binding	1
%drug binding	1
%glycosaminoglycan binding	12
%isoprenoid binding	1
%lipid binding	23
%metal ion binding	103
%nucleic acid binding	208
%nucleotide binding	132
%peptide binding	3
%protein binding	112
%pyridoxal phosphate binding	1
%receptor binding	17
%selenium binding	1
%steroid binding	2
%tetrapyrrole binding	2
%vitamin binding	1
%catalytic activity	358
%helicase activity	15
%hydrolase activity	162
%integrase activity	1
%isomerase activity	22
%kinase activity	28
%ligase activity	22
%lyase activity	11
%oxidoreductase activity	88
%small protein activating enzyme activity	2
%small protein conjugating enzyme activity	8
%transferase activity	59
%transposase activity	2
%chaperone activity	23
%heat shock protein activity	7
%enzyme regulator activity	30
%caspase regulator activity	1
%enzyme activator activity	10
%enzyme inhibitor activity	16
%GTPase regulator activity	9
%kinase regulator activity	3
%nitric-oxide synthase regulator activity	1
%molecular_function unknown	29
%motor activity	11
%microtubule motor activity	2
%signal transducer activity	54
%receptor activity	26
%receptor binding	17
%receptor signaling protein activity	4
%structural molecule activity	160
%extracellular matrix structural constituent	2
%structural constituent of bone	1
%structural constituent of cytoskeleton	10
%structural constituent of eye lens	2
%structural constituent of muscle	2
%structural constituent of ribosome	103
%transcription regulator activity	42
%RNA polymerase I transcription factor activity	1
%RNA polymerase II transcription factor activity	5
%transcription cofactor activity	11
%transcription factor activity	24
%transcriptional repressor activity	3
%translation regulator activity	21
%translation factor activity, nucleic acid binding	21
%transporter activity	156
%amine/polyamine transporter activity	2
%auxiliary transport protein activity	1
%carbohydrate transporter activity	1
%carrier activity	66
%channel/pore class transporter activity	8
%drug transporter activity	1
%electron transporter activity	34
%intracellular transporter activity	3
%ion transporter activity	58
%lipid transporter activity	5
%neurotransmitter transporter activity	2
%organic acid transporter activity	3
%oxygen transporter activity	6
%peptide transporter activity	2
%protein transporter activity	33

^a ^A selection of GO categories is presented. All annotations are accessible at

^b ^Indented terms are 'children' of the above 'parent' term.

Table 3 shows that the sequences can be classified in 12 of the major categories for molecular function, the two largest groups being catalytic activity (26%) and binding (36%).

## Discussion

The total number of gill and intestine clones available was 8256. Table 1 shows the sampled means of the insert sizes after fractionation, which varied between 700 and 1500 base pairs. cDNA cloning rarely produces full-length gene products but rather products containing varying proportions of the whole sequence. 5'sequencing may overcome this problem since clustering can be used to cover larger parts of the transcripts. In this study, after trimming and quality control followed by clustering, approximately 65% of the trimmed sequences from both libraries ended up in contigs.

Sequence annotation was carried out using GO terms. A fairly strict criterion of E-value below 1.0 × 10^-15 ^for a significant hit was used both for Blastx run against the Swiss-Prot and nr protein databases, and blastn against the nr nucleotide database. This rather low value was chosen to reduce the number of hits on very remote homologous genes with functions unrelated or only remotely related to the sequences in the current libraries. For blastx against the PDB database, the significance level chosen was an E-value below 1.0 × 10^-10^, and 489 or 16% of the sequences gave significant hits in this database. Almost all of these showed sequence similarity above 30%, indicating that they might be suitable targets for homology modelling of protein 3D structure if the hit produces a biologically meaningful alignment.

**Figure 1:**
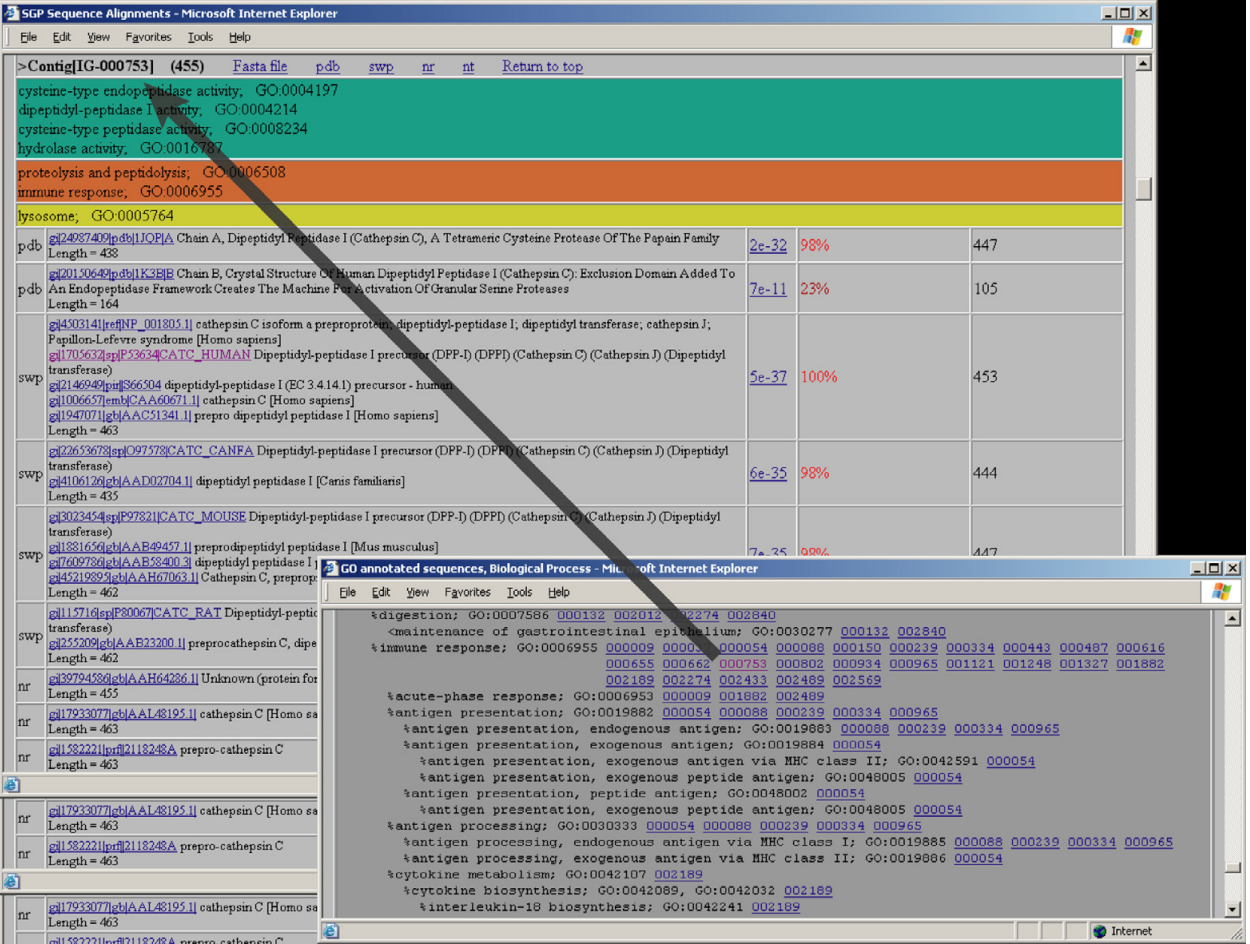
**Gene Ontology Annotation**. The annotated sequences can be reached through three documents corresponding to molecular function, biological process, and cellular component GO terms. There are links to all results for a given GO term -"immune response" is used in the figure – that can be followed to the sequence information and alignment summary for each sequence. The main tables give links to the fasta file containing the sequence itself and the BLAST results for each sequence. The most significant sequence alignment results are also given with various details such as expectation value and length of sequence alignment hit. See the web site for further details.

Approximately one third of the sequences gave hits when blastx was run against the Swiss-Prot database, and were annotated by annotation transfer from GO-annotated UniProt sequences as described in section 2.6. Since we used a fairly strict criterion for a significant hit, we would argue that the derived GO annotation is meaningful for a large fraction of these 1005 sequences. We plan to use a more elaborate annotation pipeline for future work, including estimates of accuracy and sensitivity and making it possible to detect more remote homologous sequences, for example by profile-based sequence similarity searches. Annotating the sequences and collecting the links to all annotated sequences in tables that correspond to molecular function, biological process, and cellular component GO terms makes it possible in future studies to extract genes of interest merely by looking at the GO term tables from the links at the Salmon Genome Project website [11] in the Annotation section (Data and Results > Annotations > SGP Intestine-Gills). Figure 1 illustrates this. A researcher interested in immunology would simply go to the biological process GO term table and search for 'immune response', and could then follow each of the 25 links to the sequences that appear to have a connection with immunology and their sequence alignment results. This will make it possible to find possibly relevant sequences much more directly, without having to browse through thousands of sequences and their associated sequence alignment data.

In the two libraries, we discovered several genes that may be involved in the smoltification process, genes involved in cell homeostasis and genes coding for hormones that may influence salmon maturation. Certain genes were of particular interest, for example the glucagon-family neuropeptide precursor, tyrosine 3/ tryptophan 5-monooxygenase activities and thyroid receptor interactin protein 6 (TRIP6). In addition, five different genes related to growth factor activity were also present: granulin, neuregulin 1, syntenin, Bmp2 and pleiotrohin 1. All of the above-mentioned genes are thought to play a part in the smoltification process in salmon, along with the osmoregulatory genes. However, it is possible that some of the more interesting genes involved in the smoltification process have not yet been annotated and characterised and are therefore listed as unknown. Approximately 50% of the sequences gave no significant hits in the blastx-blastn pipeline. A large proportion of these gave hits with higher E-values to more remote homologous sequences, but a significant number, at least 500–1000 sequences, are of completely unknown function at present. However, the 5' UTR of fish may differ significantly from that of mammals and terrestrial organisms. This means that the failure to match 500–1000 sequences does not confirm that the genes are unique to fish or markedly different from the coding region of transcripts of other organisms. A number of factors, including the 5'UTR, may help to explain why no similarity was found for these sequences, but the unusual biology of salmon and the fact that the sequences are from pre-smolt fish may be contributory factors. Pre-smolt salmon live in fresh water, and presumably different sets of genes are turned on and off in response to the two different habitats, the freshwater and the marine environment. In addition, the lack of similarity to already known sequences in other vertebrates could influence these results. Although large amounts of sequence data from other species have been generated, a large fraction of the genes in higher organisms is still uncharacterised. It therefore seems likely that the lack of similarity of these ESTs to known genes is partly explained by the fact that many genes are still uncharacterised.

## Conclusion

This work has made available approximately 3000 sequences from pre-smolt salmon, one third of which have been annotated for function, biological process and cellular component. Several hundred sequences code for proteins that show enough sequence-similarity to proteins of known structure to be suitable candidates for homology modelling. The results of this study will be a valuable resource for future studies of Atlantic salmon biology since it is now possible to search for genes sequenced in salmon rather than having to use comparative data from other species.

## Methods

### 1. Construction of cDNA libraries

Pre-smolt Atlantic salmon (Salmo salar) from a commercial aquaculture-bred stock (National Norwegian Breeding Programme for salmon, Norway), weighing 50–100 g, were collected in late September for the construction of two cDNA libraries from the intestine and gills. The entire intestine and the whole of the gills were used to construct the libraries. Tissue samples of 400 mg were used for total RNA extraction, which was performed using the guanidine thiocyanate-phenol-chloroform extraction method [12]. mRNA was further isolated using the Poly (A) Quick^® ^mRNA isolation kit (Stratagene Cloning Systems, California, USA). Three μg of total RNA and 0.1 μg of mRNA were run on an agarose gel to investigate the integrity/quality of the samples. cDNA synthesis was performed using the pBluescript^® ^II XR cDNA library construction kit (Stratagene Cloning Systems, California, USA), which generates directional cDNA libraries using an oligo (dT)_18 _primer. For each library the cDNA was size fractionated using a gel filtration column, and three fractions containing most of the cDNA were ligated into the plasmid vector and subsequently transformed into XL10 Gold ultra competent cells.

### 2. Pre-screening of the cDNA libraries

Pre-screening was performed to separate abundant genes from rare ones. From each of the three cDNA fractions from both tissues, approximately 50 000 colonies were plated out on agar plates (140 mm in diameter), with an average of 2000 colonies per plate. The colonies were transferred to 132 mm BIOTRANS nylon membranes, pore size 1.2 Micron, before hybridisation to total cDNA. Total cDNA from the intestine was used to screen the intestine filters and total cDNA from the gills to screen the gill filters. The cDNA was labelled using α^32^P-dCTP in a PCR assay. In order not to label the vector, the plasmids containing the cDNA were digested with XhoI/EcoRI to open up the circle. Then M13–20 primer was used in a 'one way' PCR to label the insert. The colonies that hybridised were presumed to be the more abundant ones in the libraries. A total of 3840 non-hybridising clones (the presumed rare genes) and 288 hybridising clones were picked from each of the gill and intestine libraries and gridded in duplicate into 384 well microtiterplates. The plates were grown overnight in LB, then glycerol was added and the plates were sealed and stored at -80°C.

### 3. Enrichment of pBluescript cDNA libraries

The remaining cDNA was amplified in semi-solid fashion, as described by the manufacturer (Stratagene Cloning Systems, California, USA). Amplification in suspension allowed three-dimensional, uniform growth, which reduced the likelihood of under-representation of particular clones. Up to 5 × 10^5 ^cfu/bottle of primary library was added to each bottle of the semi-solid suspension, before incubation on an ice-water bath for an hour to achieve the required semi-solid state of the medium, followed by incubation for 40–45 hours at 30°C. Afterwards the contents of the bottles were spun down and resuspended in 50 ml of 2xLB-glycerol (12.5%) for the gill library and 100 ml for the intestine library, ending up with 8 × 10^9 ^clones from the gills and 3.1 × 10^10 ^clones from the intestine. Total volumes of 1 ml were aliquoted into tubes and subsequently frozen at -80°C for future use.

### 4. DNA isolation procedures

A few microlitres of the pre-screened cDNA library was cultured overnight in LB before the DNA was isolated using the QIAprep 96 Turbo Miniprep kit from QIAGEN in a QIAvac96 according to the manufacturer's manual.

### 5. Size determination of the library fractions

To determine the insert sizes of the clones from the different size fractions, a rough estimate was made for 192 randomly picked clones from each fraction. The insert size was determined by digesting the insert out of the vector using the restriction enzymes EcoRI and XhoI, followed by separation on an agarose gel. The gels were scanned on a Typhoon 9410 Imager and analysed using the ImageQuant TL software, both from Amersham Biosciences.

### 6. DNA sequencing

Sequencing was done from the 5'-end using T3 as sequencing primer. 5'-sequencing was chosen in order to assign functional annotation to as many transcripts as possible. The sequencing reactions were performed using the ABI PRISM^® ^BigDye™ Terminators Cycle Sequencing Kit (Applied Biosystems), and run on the ABI 377 (Applied Biosystems) or on the Megabace 1000 (Amersham Pharmacia).

### 7. Clustering, sequence comparison and annotation transfer

Phred [13,14] and cross_match (Green P: unpublished) were used for basecalling and trimming of vector, respectively. The sequences were masked for repeats against a Danio rerio repeat library [15], and sequences containing contaminants or that were of poor quality were removed. The remaining high quality sequences were assembled and clustered with the Sequencher 4.1 package. In order to examine sequence similarity to known genes, NCBI blast sequence alignment [16] was performed against the NCBI databases [17] (on 08.03.2004). For each sequence, blastx was run against the PDB, Swiss-Prot and nr protein sequence databases, and blastn was run against the nr nucleotide sequence database. We defined a significant database hit as having an expectation value (E-value) below 1.0 × 10^-15 ^for all sequence alignments, except for blastx against PDB where 1.0 × 10^-10 ^was used. All sequences that gave a significant blastx hit in Swiss-Prot were annotated by annotation transfer, applying the Gene Ontology (GO) [18,19] assignments for the UniProt database produced by the GOA project of the European Bioinformatics Institute[20]. The gene_association.goa_uniprot database of 26.04.2004 was used [21] together with GO terms from the GO release of 11.05.2004 [22]. The sequences were annotated on the basis of the single best hit in the Swiss-Prot database.

## Authors' contributions

HHL performed the experiments and drafted the manuscript. JKL participated in the sequence comparisons, carried out the annotation transfer, designed the web pages and drafted the bioinformatics section of the manuscript. FP carried out the sequence alignments on the entire SALGENE sequences. AA participated in the sequence comparisons and provided supervision on the bioinformatics section. BH conceived the study, participated in its design and coordination, provided supervision and helped to draft the manuscript. All authors have read and approved the final manuscript.
